# Spinal subarachnoid haemorrhage secondary to spinal rheumatoid vasculitis: a case report

**DOI:** 10.1186/s12883-021-02497-x

**Published:** 2021-11-30

**Authors:** Yeqing Xiao, Jie Yang, Jian Xia, Yunhai Liu, Qing Huang, Jie Feng

**Affiliations:** Department of Neurology, Xiangya Hospital, Central South University, Xiangya Road 87, Changsha, 410008 Hunan Province China

**Keywords:** Case report, Spinal vasculitis, Rheumatoid arthritis, Rheumatoid vasculitis, Spinal subarachnoid haemorrhage

## Abstract

**Introduction:**

Spinal subarachnoid haemorrhage is extremely rare in cases of subarachnoid haemorrhage and possesses servere characteristics. Additionally, spinal rheumatoid vasculitis is rare for spinal subarachnoid haemorrhage. The pathogenesis is unknown.

**Case presentation:**

A 52-year-old woman with a 10-year history of seropositive rheumatoid arthritis was managed with leflunomide and celecoxib, and stable low disease activity was achieved. The patient had also been diagnosed with spinal subarachnoid haemorrhage secondary to isolated spinal rheumatoid vasculitis and obtained good therapeutic effects.

**Conclusion:**

This is the first case to describe spinal subarachnoid haemorrhage secondary to isolated spinal vasculitis in a patient with rheumatoid arthritis, which provides more proof of anomalous neovascularization in the central nervous system in rheumatoid arthritis.

## Introduction

This case reports on a spinal subarachnoid haemorrhage (SAH) secondary to isolated spinal vasculitis in a patient with rheumatoid arthritis. SAH is a neurologic emergency [1], and spinal SAH is rare in all cases of SAH [2]. It is most commonly found in association with spinal cord trauma [1]. Many other causes of spinal SAH unrelated to trauma include arterial aneurysm, arteriovenous malformation (AVM), arteriovenous fistula, vasculitis, connective tissue disease, and tumours [3]. Rheumatoid vasculitis (RV) is extremely rare but life-threatening disease in rheumatoid arthritis (RA). Moreover, cerebral nervous system vasculitis is extremely rare and is associated with male patients with incidences of long-standing, nodules, smoking, and erosive positive rheumatoid factor [4]. Spinal SAH associated with RV has rarely been reported, and the mechanism is unknown. The objective of this case was to describe spinal SAH secondary to isolated spinal vasculitis in a patient with RA.

## Patient information

A 52-year-old woman was admitted to the medical centre for sudden chest and back pain for 14 days; additionally, she was aggravated with weaknesses of both lower limbs, and she had stool and urination disorders for 9 days. She had a history of seropositive RA for more than 10 years, which was managed with leflunomide and celecoxib, whereby stable low disease activity had been achieved. The patient had also been diagnosed with hypertension grade 1 approximately 0.5 months ago. No family medical history was found.

## Clinical findings

### Physical examinations

She had limb joint deformation and swan-neck deformities; additionally, the muscle strength of the upper limbs was normal, where it was approximately 3**–**4/5 in the lower limbs. The bilateral Babinski sign and Hoffman sign were negative. There were no obvious abnormalities in bathesthesia and superficial sensation. Decreased tendon reflexes and muscle tone of lower limbs, constipation, bladder and sphincter dysfunction, Meningeal symptoms of nuchal rigidity, Brudzinsky and Kernig signs was positive.

### Laboratory and imaging data

Laboratory investigation indicated mild anaemia and elevations of leucocytes, interleukin 1 beta, tumor necrosis factor alpha. Additionally, she was exhibited persistently elevated erythrocyte sedimentation rate and C-reactive protein, as well as the presences of positive rheumatoid factor (RF), anti-cyclic citrullinated peptide (CCP) antibodies, positive anticardiolipin antibody M, and anti-nuclear antibodies. A cerebrospinal fluid (CSF) examination showed bloody cerebrospinal fluid and elevated microprotein (4.71 g/L). The following examinations were negative: anti-Sjogren**’**s syndrome A and B antibodies, preliminary screening of LA1 for lupus anticoagulant, antinuclear antibody assay, complete set of lupus, antibody of anti-beta2-glycoprotein, examination related to vasculitis, tuberculosis antibody, skin testing of tuberculin pure protein derivative experiment and T-spot experiment.

**Fig. 1:**
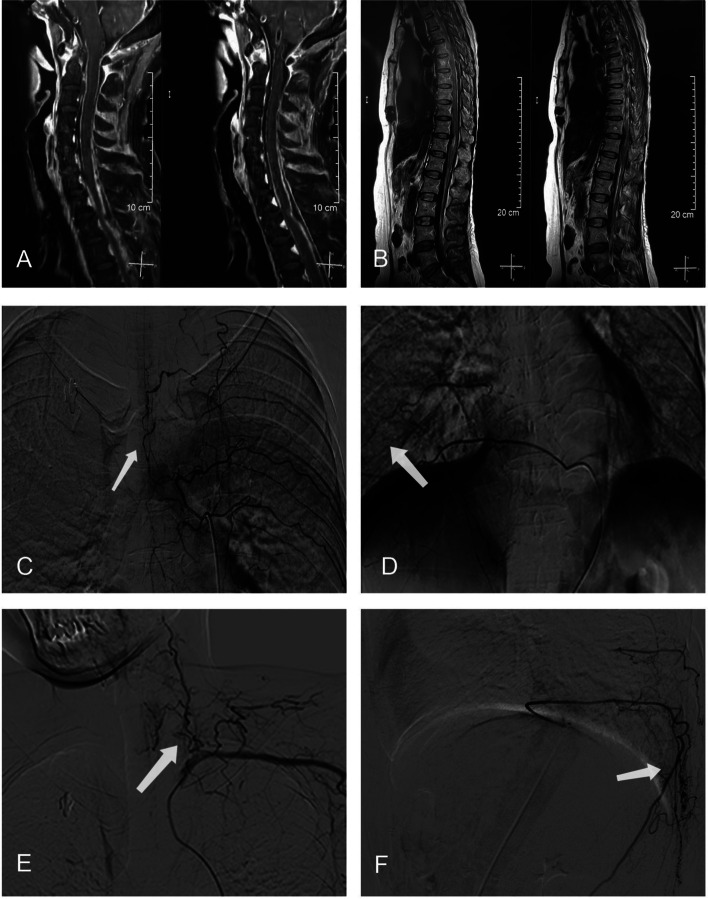
Spinal cord MRI showed subarachnoid haemorrhage in the cervicothorax and waist segments (**A** and **B**). Digital subtraction angiography showed beaded changes of spinal cord vessels in the thoracic spinal cord and lower cervical spinal cord (**C**, **D**, **E** and **F**) (arrows)

A chest scan showed multiple inflammation nodules. Joint radiographs of the limbs showed that the joints of the bilateral hands and feet were deformed. Electromyography revealed that the motion amplitudes of the upper and lower limbs were low. Specifically, the lower left limb somatosensory evoked potentials showed suspicious central damage; and abnormal skin sympathetic response in both lower extremities. Magnetic resonance imaging (MRI) of the spinal cord showed subarachnoid haemorrhage of the spinal cord, and digital subtraction angiography (DSA) showed beaded changes in spinal cord vessels in the thoracic spinal cord; additionally, the lower cervical spinal cord suggested vasculitis (Fig**.** 1). No obvious abnormalities were found in the vascular ultrasound of the extremities, carotid duplex ultrasound, or intracranial magnetic resonance imaging angiography.

## Diagnostic assessment

She was diagnosed with RA according to the European League Against Rheumatism (EULAR) recommendations for the management of rheumatoid arthritis (2016). Except for vasculitis, she suffered from additional symptoms, including mild anaemia and multiple inflammation nodules. According to the previously mentioned data, juvenal rheumatic arthritis, osteoarthritis, and ankylosing spondylitis, among other disorders, were excluded. In addition, her spinal computed tomography angiography (CTA) showed spinal cord SHA, which was supported by her clinical features. Her spinal DSA showed that the vasculitis manifestation in the spinal vasculature may be the reason for the SHA. However, it challenging to distinguish the source of the haemorrhage from an isolated small aneurysm by the use of angiography.

## Therapeutic intervention and follow-up

Upon diagnosis, she was asked to perform complete bedrest. She accepted the associated treatment for SHA and avoided complications. In addition, she received treatments with MTX and prednisone. After a week, she had obvious recovery of limbs weakness and constipation and urinary retention. At the follow-up of a half month, she did not exhibit relapses. Further follow-up was continued.

## Discussion

Spontaneous spinal subarachnoid haemorrhage is extremely rare and occurs in only < 1% of all cases of SAH [2]. Approximately 1% of all cases of SAH in the spinal cord are caused by a primary spinal cord aetiology [5]. In addition, few cases of central nervous system (CNS) rheumatoid arthritis have been reported [6]. In this case, the female patient presented with RA with long-standing, nodules and an erosive positive rheumatoid factor diagnosis of SAH of the spinal cord. It is most likely that the SAH observed in this case was the result of rheumatoid vasculitis. Thus far, this case of SAH in the spinal cord due to CNS rheumatoid vasculitis has not been described in the literature. In fact, it is easily to misdiagnosed as ruptures of spinal aneurysms or other causes, and spinal SAH may be especially difficult, as it may lead to cerebral symptoms.

RV has been reported at a high rate in patients with RA. The incidence rate of RV has decreased since 1990, as has been proven by recent studies, but the lethality rate is high [7]. RV is often involved in cerebral, mesenteric, and coronary arteries [8]. Vasculitis involving the cerebral nervous system is extremely rare. Previous studies have reported CNS vasculitis in the cerebral vasculature and dura mater; additionally, CNS vasculitis has been much less reported in the leptomeninges and choroid plexus, and it is extremely rare in the cerebral parenchyma [9]. RV involving the CNS may be in a type of systematic or isolated state in patients with RA [6]. Some studies have suggested that CNS vasculitis is a part of RA, or it may even be a comorbidity [9]. The prevalence of RV involved in the CNS is approximately 0.5–1% worldwide, and it is mostly observed in middle-aged women [9]. Moreover, few cases of central nervous system vasculitis are associated with positive anti-neutrophilic cytoplasmic antibodies or systemic lupus erythaematosus [10].

In conclusion, RV is uncommon in the CNS, and rarely leads to SAH. The course and onset patterns of SAH secondary to RV of the CNS are easily misdiagnosed as ruptures of spinal aneurysms. Therefore, the timely improvement of spinal cord vasculitis DSA is vital for further identifications of the true causes of SAH. In a word, this case reported spinal subarachnoid haemorrhage secondary to isolated rheumatoid vasculitis in the spinal in a patient with rheumatoid arthritis, which provides more details on the vasculature involved in the CNS in RA. In this case non operative management was a good option. What’s more, the adverse effect of leflunomide as a cause of vasculitis, which could be assumed as an etiology in future research about the topic.

## Data Availability

All data generated or analyzed during this study are included in this published article.

## Abbreviations

SAH: Subarachnoid haemorrhage
AVM: Arteriovenous malformation
RV: Rheumatoid vasculitis
RA: Rheumatoid arthritis
RF: Positive rheumatoid factor
CCP: Anti-cyclic citrullinated peptide
CSF: Cerebrospinal fluid
MRI: Magnetic resonance imaging
DSA: Digital subtraction angiography
EULAR: European League Against Rheumatism
CTA: Computed tomography angiography
CNS: Central nervous system
